# Prevalence of cancer in relation to signs of periodontal inflammation

**DOI:** 10.1371/journal.pone.0276375

**Published:** 2022-10-21

**Authors:** Jukka H. Meurman, Håkan Källmén, Leif. C. Andersson, Tulay Yucel-Lindberg, Birgitta Söder

**Affiliations:** 1 Department of Oral and Maxillofacial Diseases, University of Helsinki and Helsinki University Hospital, Helsinki, Finland; 2 Center for Psychiatry Research Department of Clinical Neurosciences, Karolinska Institutet, Stockholm, Sweden; 3 Department of Pathology, University of Helsinki and Helsinki University Hospital, Helsinki, Finland; 4 Department of Dental Medicine, Karolinska Institutet, Stockholm, Sweden; Klinikum der Johann Wolfgang Goethe-Universitat Frankfurt Klinik fur Nuklearmedizin, GERMANY

## Abstract

We investigated the associations between periodontal inflammation (gingivitis and periodontitis) and all-kind malignancies, specifically breast and prostate cancer, in a cohort followed-up for 30 years. The study hypothesis was based on the oral inflammation *vs*. systemic health paradigm. A sample of 2,168 subjects from an original cohort of 105,718 individuals from the greater Stockholm area in Sweden that had been followed since 1985 was investigated. Swedish national health registers were used in the study. Chi-square tests and logistic multiple regression analyses were conducted. The results showed that periodontitis was significantly associated with any cancer after adjusting for gender, age, income, and education (p = 0.015). The probability of getting cancer increased on average by 38% if the patient had periodontitis *vs*. had not; the odds ratio was 1.380 (95% confidence interval l.066-1.786). No significant association was observed between periodontitis and breast cancer (p = 0.608), while the association between periodontitis and prostate cancer tended towards significance (p = 0.082). However, no statistically significant difference was found between the observed and the calculated distribution of any cancer in gingivitis groups (p = 0.079). Thus, the study hypothesis was partly confirmed by showing a statistically significant association between periodontitis and any cancer.

## Introduction

Infection and inflammation link to about 15–20% of all malignancies. Even 20% of cancer deaths have been estimated to associate with chronic infections and persistent inflammations [1]. The pathogenic mechanisms involved are microbiota-caused upregulation of a number of cytokines and other inflammatory mediators that may also affect DNA repair mechanisms [2]. The mouth harbors hundreds of microbial species and pathogenic bacteria like the periodontitis-associated pathogen *Porphyromonas gingivalis*. Periodontitis, on the other hand, affects 15–35% of the adults in industrialized countries and there is considerable variation between coutries [3, 4]. This long-term infection leads to continuous low-grade bacterial invasion in the bloodstream with subsequent systemic consequences in other organs [5]. Oral infections have previously been statistically linked to cancer [2, 6].

Breast cancer is the most prevalent cancer in women, while prostate cancer is the most common cancer in men [7]. In autopsy reports, prostate cancer is a frequent finding presenting 30% in >50 year-olds and up to 80% in > 80-year-olds. High age is the principal risk factor for prostate cancer [8]. In addition to age, the American Cancer Society [9] lists the following prostate cancer risk factors: race and ethnicity (in particular in African-American men), geography (most common in North America, Europe, and Australia), family history, gene changes (especially BRCA2 mutations), diet (high consumption of red meat and high-fat products), obesity, smoking, exposure to chemicals, and prostatitis.

Karan & Dubey [10] reviewed the role of inflammation in the development of prostate cancer and concluded that it might affect pathogenesis. Inflammation of the prostate, *i*.*e*., prostatitis, has been extensively studied in this respect, but the results are contradictory. For example, a screening study conducted in Finland, where inflammation was analyzed from prostate biopsy specimens in men with high serum prostate-specific antigen values, failed to show that histological signs of local inflammation would link to an increased risk of prostate cancer [11].

As said, in women, breast cancer is the number one malignancy worldwide [12]. Here, too, the infection has been suggested to play a role in the pathogenesis of breast cancer [13]. Interestingly, during the covid-19 pandemic, the virus has been shown to enhance the metastatic spread of breast cancer [14]. In earlier studies, human papillomaviruses, in particular have been investigated in this respect, but causality has not been confirmed [15]. We have previously investigated the association of periodontitis with breast cancer in our long-term follow-up investigation and observed that if a woman had periodontal disease and any missing molars in the mandible, 5.5% had breast cancer in comparison to 0.5% of the subjects who had a periodontal disease but no missing molars in the mandible [16]. Missing molars can be regarded as a proxy for earlier oral infection because molar teeth are usually not extracted unless they are infected. Thus, the finding supported the focal infection theory.

Hence, inflammation in and around cancer tissue may affect carcinogenesis [17]. In this perspective, the role of tissue-specific inflammasomes has been discussed. These protein molecules are major regulators of inflammation, leading to up-regulated expression of a number of cytokines and inflammatory mediators such as interleukin (IL)-1β and IL-18 [18]. In general, inflammation has been associated with many kinds of cancers, including head and neck cancer [19, 20].

In the present register study, we investigated the prevalence of all-kind malignancies concerning the patients´periodontal status, gingivitis, and periodontitis data. The focus was on the most prevalent cancers, namely breast cancer in women and prostate cancer in men. We anticipated that periodontal disease would associate with cancer. The hypothesis was based on the oral inflammation—systemic health paradigm.

## Material and methods

### The cohort

**Fig 1:**
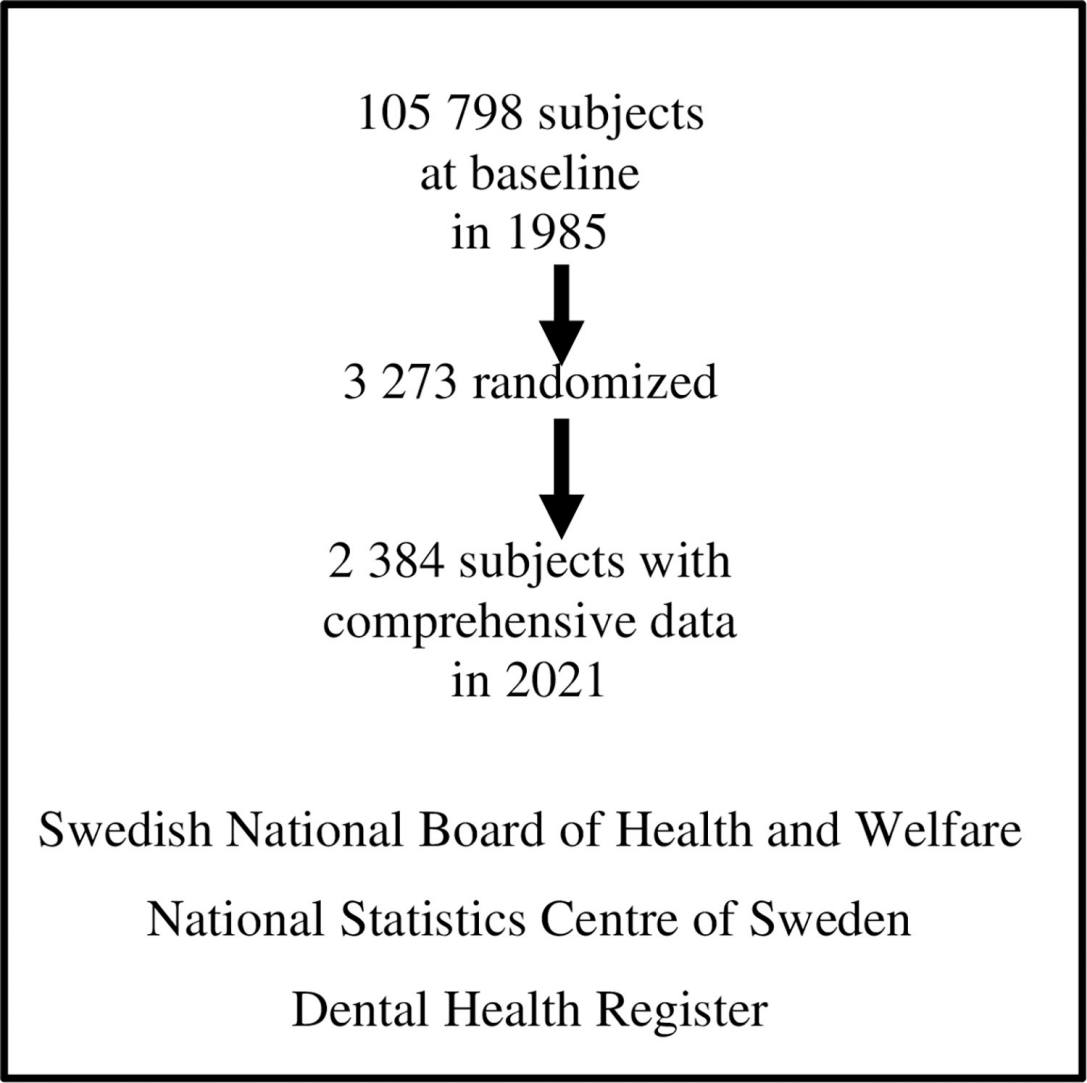
Study profile and the national registers.

This database study originates from the year 1985 when altogether 3273 randomized subjects were invited from the Stockholm metropolitan area, Sweden, to participate in a clinical investigation with a focus on periodontal health. The subjects were born on the 20th day of any month from 1945 to 1954, and the size of the whole cohort was 105798 persons [21]. The subjects´ health parameters have been followed up for more than 30 years using the nationwide Swedish population and health registers. For the current study, a sample of 2384 subjects was used based on comprehensive records of their gender: 1244 women and 1140 men. Their social security number data were then linked to the dental health register (“Tandvårdsregister”), where the periodontal diagnostic codes, gingivitis and periodontitis, and frequency of dental appointments could be analysed. This resulted in 2168 subjects, 1136 women and 1032 men, respectively, for further analyses. The subjects had dental appointments from 0 to 63 times during the long follow-up, with a median of 11 appointments (interquartile range 9–16). There were 217 patients who were not identified in the dental health register and were deleted from the analyses. In the further analyses, the Swedish Cancer Register, Hospital Admission Database, and Death Register of the Center of Epidemiology from the Swedish National Board of Health and Welfare were used to find out if there were associations between the prevalence of malignancies and periodontal health parameters. The socio-economic data, such as age, education, yearly income, social status, and working status, were obtained from the National Statistics Centre, Örebro, Sweden. **Fig 1** summarizes the study profile and the registers used for analyses.

For statistical analyses, all cancer diagnoses recorded with the WHO ICD-10 codes (World Health Organization International Classification of Diseases-10) were extracted from registered files and included in a variable “any cancer”. It was dichotomized and coded one if any cancer was present and 0 if not present. In addition, dichotomized variables “breast cancer” and “prostate cancer” were also constructed and coded similarly. Age was calculated from the 2017 registers where also the cancer data were available. This resulted in an age range of 62–72 years (median 67 years). For analyses, the age variable was also dichotomized so that up to age 67 years, it was coded as one, while for age 68 years and more, the code was 2. The dichotomized age variable was then used as a control variable in the analyses.

### Statistical analyses

First, a cross-table was made where gingivitis and periodontitis were calculated against cancer and tested with the Chi-square test. If the Chi-square was significant a multivariable logistic regression analysis was thereafter conducted to analyze the associations between the inflammatory variables, gingivitis, and periodontitis, with the prevalence of cancer by controlling for gender, age, and socio-economic status. Data on prostate cancer and breast cancer were similarly analyzed. SPSS 26 software program was used. Odds ratios were derived from the regression, and in the case of negative beta, the inverse of odds ratios was calculated to estimate the increased risk of cancer.

### Ethics

The Ethics Committee had approved the study of the Karolinska University Hospital at Huddinge (Dnr 2007/1669–31; 2012/590–32; 2017/2204–32). A written informed consent was obtained.

## Results

The prevalence of gingivitis and periodontitis, obtained by diagnosis codes, was about 70% in men and women in this sample, shown in **Table 1**. The corresponding prevalences of breast and prostate cancer were 7.9% and 7.2%, respectively. The prevalences of any cancer for men and women were 15.2% and 20.4%, respectively. There was no statistically significant difference between the observed and the expected distribution in getting any cancer between patients with or without gingivitis during the time of observation (p = 0.079). But there was a difference between those with cancer who had or had not periodontitis (p = 0.009). Results from logistic regression analysis, given in **Table 2**, showed that periodontitis was significantly associated with any cancer and that the probability of getting cancer increased on average by 38% if the patient had periodontitis *vs*. had not. The analysis was controlled by age, sex, and socio-economic status. A significant association was also found between age and any cancer. On average, patients over 67 years had a 37% higher risk than those aged 67 or younger of having cancer. Gender was significantly associated with any cancer. Women appeared to have a 46% higher risk for any cancer in general.

**Table 1:** Prevalences in percent of the 2168 men and women who had the cancer diagnoses breast cancer and prostate cancer and the periodontal inflammation gingivitis and periodontitis.

Gender	Gingivitis	Periodontitis	Breast cancer	Prostate cancer
Male (n = 1032, 47.6%)	74.1	73.4	0	7.2
Female (n = 1136, 52.4%)	78.1	68.7	7.9	0

**Table 2:** Results from logistic regression analysis on periodontitis *vs*. any cancer. Beta coefficient and standard error, Walds chi sqare and p-value, odds ratio and 95 percent confidence interval (CI).

Variable	Wald	B	SE	p-value	OR (95% CI)
Periodontitis	5.976	0.322	0.132	0.015	1.380 (1.066–1.786)
Sex	10.844	-0.381	0.116	0.001	0.683 (0.544–0.857)
Education	0.549	-0.087	0.117	0.459	0.917 (0.729–1.153)
Income	0.244	0.058	0.118	0.621	1.060 (0.842–1.334)
Age	22.482	0.539	0.114	0.000	1.372 (1.372–2.141)
Constant	112.422	-2.377	0.224	0.000	

When analyzing the association between gingivitis and breast cancer, no statistically significant result was obtained (**Table 1**). Altogether, 1651 patients had gingivitis while 516 had not. Only 18 patients with breast cancer had no gingivitis, while 72 breast cancer patients did have gingivitis (p = 0.366). As regards the observations on the association between periodontitis and breast cancer, 24 breast cancer patients did not have periodontitis while 66 patients with breast cancer had periodontitis; Chi-square was not significant, however (P = 0.608). The results from the logistic regression analysis are given in **Table 3**.

**Table 3:** Results from logistic regression analysis on periodontitis *vs*. breast cancer. Walds chi-sqare and p-value, odds ratio and 95 percent confidence interval (CI).

Variable	Wald	B	SE	p-value	OR (95% CI)
Periodontitis	0.263	0.125	0.243	0.608	1.133 (0.703–1.825)
Constant	240.676	-3.229	0.208	0.000	0.40

Data on prostate cancer showed that 58 patients with gingivitis had prostate cancer diagnostic code while 16 had not (ICD-10 code C61). This difference was not statistically significant (Chi-square, P = 0.653). As regards data on periodontitis and prostate cancer, a tendency toward significant difference could be seen (P = 0.067). Of the 757 patients who hadperiodontitis, 61 patients (8.1%) had prostate cancer while among the 275 periodontally healthy 13 (4.7%) had prostate cancer.The results from multivariable logistic regression analysis are given in **Table 4**. There was a non-significant tendency (p = 0.082) to an association between periodontitis and prostate cancer after controlling for education, income above the median, and age. Both income and age were significantly associated with prostate cancer. Age equal to or above 67 years compared with that below, more than doubled the risk on average to also have prostate cancer. Further, income above the median compared with that below increased the moderate risk for prostate cancer by 74%.

**Table 4:** Results from logistic regression analysis on periodontitis *vs*. prostate cancer. Beta and standard error, Walds chi-sqare and p-value, odds ratio and 95 percent confidence interval (CI).

Variable	Wald	B	SE	p-value	OR (95% CI)
Periodontitis	3.019	0.550	0.317	0.082	1.734 (0.932–3.227)
Education	1.167	-0.274	0.254	0.280	0.760 (0.463–1.250)
Income	4.381	0.557	0.266	0.036	1.745 (1.036–2.940)
Age	10.539	0.818	0.252	0.001	2.265 (1.383–3.710)
Constant	67.548	-4.464	0.543	0.000	0.012

## Discussion

This investigation aimed to analyze possible associations between two oral inflammation sources, gingivitis and periodontitis, and the incidence of cancer based on the hypothesis that long-term, low-grade inflammation is a risk for malignant transformation. The data were derived from our over 30-years of observation of a cohort and registered data from Sweden. First, we investigated the associations with any cancer and, secondly, specifically with breast cancer and prostate cancer, respectively.

Our hypothesis was partly confirmed. The results showed that periodontitis seemed to pose a risk for the development of any cancer also when controlled for gender, age, and socioeconomic status. This is in agreement with the report by Meurman and Bascones-Martinez [2], and it is also partly confirmed by findings from our earlier investigation [16]. But the associations were not significant when breast cancer and prostate cancer were separately analyzed, which is partly against our findings ten years earlier from the same cohort [16]. This may be due to the high prevalence of oral inflammation in the sample, which was more than double the prevalence as has been earlier reported by Socransky et al. [22]. However, our earlier study had a shorter follow-up period. It showed that among people with periodontal disease those who also had missing molars in the mandible compared to those who did not have missing molars were more than twice as likely to also have breast cancer [16]. In the present register study there are no data on missing molars and participants without periodontitis were included in the analysis. The present calculations further showed a tendency that periodontitis might pose a light risk also for prostate cancer. This seemingly speaks against the observations by Yli-Hemminki et al. [11], but it should be noticed that their conclusion was based on signs of local inflammation in the prostatic tissue. Nevertheless, in the present study, we did confirm the result of Haas et al. [8], namely that high age is a risk factor for prostate cancer as expected. No such result was found between gingivitis and cancer, however. This might be explained by the assumption that gingivitis does not cause as heavy long-term systemic inflammation as periodontitis. For example, recently Valiyaveetil and coworkers reported how serum CRP concentrations indeed reflect the severity of periodontal inflammation [23].

As said, inflammation plays a role in the development of malignancy with a number of putative pathogenic mechanisms [2, 17]. Modern research has now focused on investigating microbiome profiles with the assumption that microbial dysbiosis, in particular, could affect cancer development. This includes dysbiosis also of the oral microbiota [24]. Recently, Salachan and Sorensen [25] discussed the host-microbe interactions in prostate cancer, showing that certain microbial species are enriched while others are decreased in the prostatic and seminal fluid. *Escherichia coli* has been specifically investigated in this regard [26]. In breast cancer, on the other hand, the bacterium *Methylobacterium radiotolerans* was shown to be enriched in tumor tissue. In contrast, the bacterium *Sphingomonas yanoikuyae* was enriched in normal breast tissue in the study by Xuan et al. [27]. The authors concluded that microbial DNA evidently is present in the breast and that it may affect tumorigenesis. However, to our knowledge, there are no studies where bacteria of oral origin would have been identified in breast cancer tissue. Hence, in future studies, it might be interesting to specifically analyze periodontal bacteria, for example, with the next generation molecular methods. Namely, periodontitis may indeed pose a risk for breast cancer [28]. We also have earlier reported this in our study from the same patient material as here [16].

As regards prostate cancer, periodontitis has been discussed as also linking to this malignancy [29]. A nationwide cohort study from South Korea, similar to our present study, showed that having periodontitis caused a 24% higher risk of prostate cancer [30]. In our study of 30 years, the result showed an average of 73% higher risk for prostate cancer if the patient had periodontitis. The Korean cohort included 121,240 individuals; their observation time was ten years. Alluri et al. [31] recently reported that *Fusobacterium nucleatum*, detected with PCR, was 10.3 times higher in prostatectomy sections of men with prostate cancer than in benign specimens. The finding thus supports the hypothesis that periodontitis might also link to prostate cancer.

In the present study, we focused particularly on the two most common cancers, namely breast cancer and prostate cancer. It is interesting to point out that periodontitis has indeed been associated with many malignancies like we here observed with “any cancer”. For example, an extensive study on women´s health in the United States observed an increased risk of lung cancer in periodontitis patients even after adjusting for smoking [32]. And, as said, there are also respective data on the association between periodontitis and head and neck cancer [20, 33]. However, it is evident that more investigations are called for the final conclusion about these connections. Gender differences in cancer susceptibility, respectively, also call for more investigations [34, 35].

The main strength of our study is the long observation time of the cohort. The over 30 years of follow-up is exceptional and, combined with the nationwide patient registers, adds to the reliability of data. However, we did not have clinical records other than those from the Swedish dental treatment register, which is a limitation. Hence, it is unknown how long and to what extent the patients had suffered from gingivitis and periodontitis during the 30 years. Consequently, the data in this respect are cross-sectional. Because of the epidemiological nature of the study, it was not possible to conduct repeated clinical examinations. An additional problem in this kind of study is the reliability of clinical examinations in recording the diagnoses. Different clinicians may use different criteria in spite of the national and international recommendations on how to register gingivitis and periodontitis [36]. One cannot avoid this, and there is no way to control it in epidemiological and register studies [37]. Finally, there were no data on our subjects’ smoking status in the records. It may be assumed, however, that those in our cohort used tobacco products the same way as is known by the general population of Sweden. In the year 2017 of the current register data, about 22% of Swedes older than 16 years used tobacco products daily, either smoked or using the Swedish snuff, according to the national statistics.

## Conclusion

A statistically significant association was found between periodontitis and any cancer. The main finding is interesting and offers possibilities for further hypothesis generation.

## Supporting information

S1 Data (XLSX)

S1 Questionnaire (DOCX)
